# Temporal correlation analysis between malaria and meteorological factors in Motuo County, Tibet

**DOI:** 10.1186/1475-2875-10-54

**Published:** 2011-03-04

**Authors:** Fang Huang, Shuisen Zhou, Shaosen Zhang, Hongju Wang, Linhua Tang

**Affiliations:** 1 National Institute of Parasitic Diseases, Chinese Center for Disease Control and Prevention; WHO Collaborating Centre for Malaria, Schistosomiasis and Filariasia; Laboratory of Parasite and Vector Biology, Ministry of Health, Shanghai 200025, China; 2 Linzhi Prefectural Center for Disease Control and Prevention, Linzhi 860100, China

## Abstract

**Background:**

Malaria has been endemic in Linzhi Prefecture in the Tibet Autonomous Region (TAR) over the past 20 years, especially in Motou County with a highest incidence in the country in recent years. Meteorological factors, such as rainfall, temperature and relative humidity in Motou County were unique compared to other areas in Tibet as well as other parts of China, thus the objective of this work was to analyse the temporal correlation between malaria incidence and meteorological factors in Motou County, in order to seek the particular interventions for malaria control.

**Methods:**

The meteorological and malaria data during 1986-2009 in Motuo County were studied to analyse the statistical relationship between meteorological data time series and malaria incidence data series. Temporal correlation between malaria incidence and meteorological factors were analyzed using several statistical methods. Spearman correlation analysis was conducted to examine the association between monthly malaria incidence and meteorological variables. Cross-correlation analysis of monthly malaria incidence series and monthly meteorological data time series revealed the time lag(s) of meteorological factors preceding malaria at which the series showed strongest correlation. Multiplicative seasonal auto-regressive integrated moving average (SARIMA) models were used in the cross-correlation analysis with pre-whitening which remove seasonality and auto-correlation of meteorological data series. Differenced data analysis which called inter-annual analysis was carried out to find underlying relationship between malaria data series and meteorological data series.

**Results:**

It has been revealed that meteorological variables, such as temperature, relative humidity and rainfall were the important environmental factors in the transmission of malaria. Spearman correlation analysis demonstrated relative humidity was greatest relative to malaria incidence and the correlation coefficient was 0.543(*P *< 0.01). Strong positive correlations were found for malaria incidence time series lagging one to three months behind rainfall (*r *> 0.4) and lagging zero to two months behind temperature and relative humidity (*r *> 0.5) by the cross-correlation. Correlations were weaker with pre-whitening than without. The cross-correlograms between malaria incidence and various meteorological variables were entirely different. It was fluctuated randomly for temperature but with trend for the other two factors, which showed positive correlated to malaria when lag was from 0 to 5 months and negative from 6 to 12 months. Besides, the inter-annual analysis showed strong correlation between differenced annual malaria incidence and differenced meteorological variables (annual average maximum temperature, annual average relative humidity and annual average rainfall). The correlations coefficients were -0.668 (*P *< 0.01), 0.451(*P *< 0.05) and 0.432(*P *< 0.05), respectively.

**Conclusion:**

Meteorological variables play important environmental roles in malaria transmission in Motou County. Relative humidity was the greatest influence factors, which affected the mosquito survival directly. The relationship between malaria incidence and rainfall was complex and it was not directly and linearly. The lags of temperature and relative humidity were similar and smaller than that of rainfall. Since the lags of meteorological variables affecting malaria transmission were short, it was difficult to do accurate long-term malaria incidence prediction using meteorological variables.

## Background

Malaria is one of the most severe infectious diseases that are seriously harmful to health, especially in border areas of west-south China. According to the report of National Surveillance Project [1], malaria was unstable and fluctuated in intensity both spatially and temporally in China. Linzhi Prefecture is located in the south-eastern part of the Tibet Autonomous Region (TAR) of China and Motuo County locates in south of Linzhi Prefecture shares borders with both India and Myanmar. A total of 2,296 malaria cases were reported from Linzhi Prefecture from 1986 to 2008, mostly due to *Plasmodium vivax*, and 2,227 of these cases (97.0%) were from Motuo County. In recent years, the malaria incidence of Motuo County was highest in the country because of its small population about 10,000 people.

The transmission and prevalence of malaria is influenced by many factors, amongst which (variability in) meteorological factors are considered to play a major role. With increasing weather variability and ability to forecast weather, there is an interest in developing systems for malaria forecasting that incorporate weather related factors as explanatory variables. Many studies [2-9] in various parts of the world had linked malaria time series to weather variables such as rainfall, temperature and humidity. For instance, malaria was associated with rainfall and minimum temperature (with the strength of the association varying with altitude) in Ethiopia by using polynomial distributed lag models. Rainfall and maximum temperature at a lag of four months to successfully fit a biological transmission model to malaria case data in a district in Zimbabwe. Inter-annual differences in malaria were linked to rainfall and temperature in South Africa. Nevertheless, others did not find a strong or an obvious correlation. A study[10] in Sri Lanka incorporating rainfall as a linear or non linear explanatory variable into a (seasonal) auto-regressive integrated moving average (ARIMA) model showed little improvement in malaria prediction over ARIMA models without a rainfall predictor.

Weather affects the malaria incidence mostly through its effects on both the mosquito vector and the development of the malaria parasite inside the mosquito vector. Motuo County has a semi-humid tropical climate with plenty of sunshine and rainfall. The diverse geographic landscapes and complex climate situations provide favorable breeding sites for anopheles mosquitoes, the vector of malaria. The primary crop in Motuo County is rice and most farmland was covered by paddy field, which suit for *Anopheles *larval habitats. In the recent studies[11-13] conducted in the Moutuo county of Linzhi Prefecture, the species of *Anopheles *included *An. maculatus *group, *An. peditaeniatus*, *An. barbumbrosus*, and *An. kochidonitz*. *An. pseudowillmori *was considered the sole malaria vector and the larval habitats only were paddy field and larval was not found in gullies, ponds, mountain creek and other small water-bodies.

Considering the unique and special characteristic of meteorological factors in Motuo County with higher malaria incidence, it may be speculated that the meteorological factors play major role in malaria transmission. In the present paper, the temporal relationship between meteorological factors and malaria incidence in Motuo County was investigated. A better understanding of the relationship may help to improve forecasting of changes in malaria incidence, which would shed light to public health authorities on how to effectively distribute resources for malaria control programmes at the national and provincial level.

## Methods

### Study area

**Figure 1:**
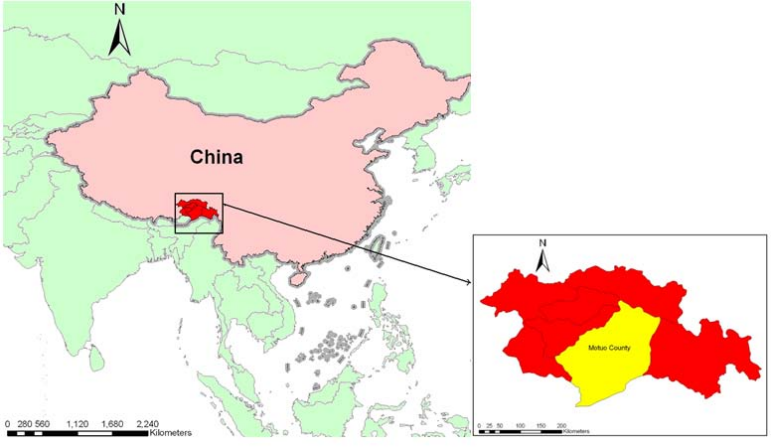
**Feature of Motuo County relative to neighboring counties and countries**.

Linzhi Prefecture locates in the southeastern part of Tibetan Autonomous Region (showed in Figure 1). The population of the prefecture amounted to 164,300 by the end of 2007 (data from Tibet Bureau of Statistics). Linzhi Prefecture has a semi-humid tropical climate with plenty of sunshine and rainfall. Motuo County is situated at 27°36'-29°50'north latitude and 93°42'-96°36'east longitude in the lower reach of Yalu Zangbu River, bordering India and Myanmar (showed in Figure 1). The altitude of the mountainous county ranges between 700 and 2,100 m (mean: 1,200 m). Up to the present day, Motuo County was the last accessible county in China, but most villages can only be reached by foot. According to the data from National Surveillance Project, almost all the malaria patients are scattered along the Brahmaputra River with spatial cluster (related analysis will be published elsewhere), where inhabited by members of the Zang, Menba and Luoba nationalities.

### Data collection and management

Data on malaria were obtained from the Center for Diseases Prevention and Control of Linzhi Prefecture, Tibet and Diseases Surveillance System of China. Monthly data of the count of blood films examined for malaria and those positive for malaria were obtained from the routinely report by the Linzhi Prefecture. Data aggregated were available for the years 1986-2009.

Population demographics for the county were provided by Tibet Bureau of Statistics. It was assumed that each resident in the county was at risk for infection of malaria. Monthly meteorological data like average temperature, average maximum temperature, average minimum temperature, relative humidity and rainfall from 1986 to 2009 in Linzhi Prefecture were obtained from China Meteorological Data Sharing Service System [14]. Temperature and rainfall variables are measured in millimeters (mm) and centigrade (°C) respectively. There are three meteorological stations surrounding Motuo County. Meteorological factors' surfaces were created through spatial prediction using Gaussian Kriging interpolation. The Gaussian Kriging interpolation was implemented in the software ARCGIS9.2. Almost all the malaria cases are centered in relative small area of Motuo County. Besides, heterogeneous altitude is not taken into account in this study. It's difficult to get the remote censoring map of the Motuo County.

### Analysis

The monthly incidence of malaria in Motuo County was treated as a dependent variable, and meteorological variables such as monthly average temperature, monthly average maximum temperature, monthly average minimum temperature, monthly average relative humidity and total amount of rainfall were independent variables. The statistical relationship between meteorological variables and malaria incidence over the period 1986-2009 in Motuo County was examined. The analyses were carried out in four aspects: 1) Spearman correlation analysis; 2) cross-correlation analysis; 3) cross-correlation analysis with pre-whitening; 4) inter-annual analysis. Since there might be auto-correlation among both dependent and independent variables over time, seasonal multiplicative auto-regressive integrated moving average (SARIMA) models with different parameters were compared on monthly time series of malaria incidence.

### Spearman correlation analysis

Spearman correlation analysis was conducted to examine the association between monthly malaria incidence and meteorological variables using SPSS software (version 17.0, SPSS Inc., Chicago, IL).

### Cross-correlation analysis

**Figure 2:**
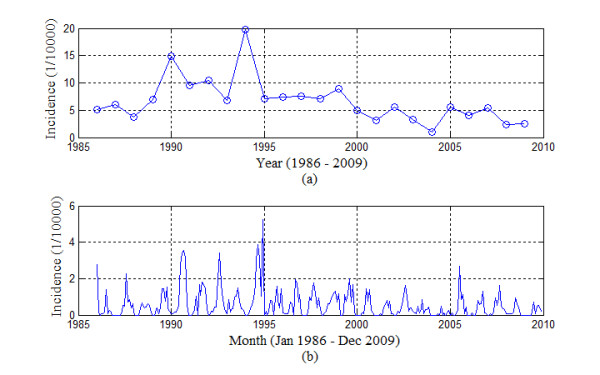
**Annual and monthly malaria incidence of Motuo County**. (a) monthly malaria incidence of Motuo County (b) annual malaria incidence Motuo County

Cross-correlations between monthly malaria incidence series and monthly meteorological data time series were analyzed to find the time lag(s) of meteorological factors preceding malaria at which the series showed strongest correlation [15-19]. Malaria incidence time series showed strong long-term fluctuations in Motuo County (Figure 2). However, in temperature, relative humidity and rainfall time series these long-term fluctuations were absent. Therefore, it was expected that meteorological variables could not explain the long-term fluctuations in malaria, which were probably related to other factors, such as malaria control measures and population changes. These fluctuations masked the correlation between malaria and meteorological variables and since no information on the underlying factors was available in the data, the long term fluctuations needed to be removed prior to calculating cross-correlations. It was assumed that monthly malaria incidence *γ_t _*follows a seasonal model of form:

$$\gamma_{t}=m_{t}+S_{t}+\varepsilon_{t},$$

Where *m_t _*is the mean level in month *t*, *S_t _*is the seasonal effect in month *t *; and *ε_t _*is the Gaussian random error.

The long-term fluctuations *m_t _*in the monthly malaria incidence series were calculated using a 13-point centered smoothing filter with the months at the extremes given half weight:

$$m_{t}=\left(0.5\gamma_{t-6}+\gamma_{t-5}+\cdots +\gamma +\cdots +\gamma_{t+5}+0.5\gamma_{t+6}\right)/12$$

Smoothing was performed using the function "decompose" of the package "stats" in the software R [20]. From the de-trended series *ξ_t _*= *γ_t _- m_t_*, implicitly long term trends were removed. Cross-correlation analysis was applied to the de-trended malaria incidence series and certain meteorological variable time series *x_t_*. The cross-correlation was estimated for malaria at a lag *l *of zero to twelve months behind rainfall as

$$r_{l}=\sum_{t=1}^{N}\left(x_{t}-\bar{x}\right)\left(\zeta_{t+1}-\bar{\zeta }\right)/Ns_{x}s_{\zeta },$$

Where *s_x_*, *s_ζ _*are the sample standard deviations of observations on *x_t _*and *ζ_t _*respectively.

The cross-correlation was calculated as the average over all months, and possible variable correlation depending on the season was not accounted for, i.e. if rainfall had a strong positive effect on malaria in some months, and a strong negative in others, the average detected cross-correlation could be weak.

Even though the above approach may find strong correlations, these may not be very useful for malaria prediction if aberrations from the long term seasonal mean of rainfall were weakly linked to aberrations from the long term seasonal mean of the malaria case series. In addition, the standard cross-correlation assumes observations were independent, whereas in reality the malaria data were temporally correlated.

### Cross-correlation analysis with pre-whitening

Cross-correlation with the seasonality and autocorrelation removed by simple pre-whitening allows for detection of the time lag(s) of meteorological variables preceding malaria, at which divergence from the long-term seasonal pattern in meteorological time series show strongest correlation with such divergence in malaria incidence time series, while minimizing effects of spurious correlations caused by autocorrelation in the time series. The effect of pre-whitening was to reduce unassociated autocorrelation and/or trends within time series prior to computation of their cross-correlation function. Simple pre-whitening was used when there was a clear unidirectional influence such as between rainfall and malaria.

First, an auto-regressive model was fit to the explanatory variable. The pre-whitened explanatory variable consisted of the residuals of this fitted model, whereas the pre-whitened outcome variable consisted of the residuals of the same model applied to the outcome variable. With the inclusion of seasonality in the autoregressive model, the pre-whitening procedure removed seasonality from the explanatory variable time series, and the same amount of seasonality from the outcome variable time series. It was thus possible that additional seasonality remained in the pre-whitened outcome variable time series.

Multiplicative seasonal auto-regressive integrated moving average (SARIMA) models [15,21] with all possible combinations of parameters *p, q, P, Q ∈{0, 1, 2}*and with d, D *∈{0, 1}*, were evaluated using the Akaike's information criterion (AIC) on untransformed and logarithmically transformed monthly meteorological data in the period from January 1986 to December 2009. The selected SARIMA model was then used to pre-whiten both meteorological data series and malaria incidence time series.

### Inter-annual analysis

Inter-annual analysis was used to analysis the correlation between the series of differenced annual malaria incidence and differenced annually meteorological data.

The difference Ω_*t,k *_= *Y_t,k _*- *Y*_*t*-1,*k *_reflects the relative change in incidence between consecutive years [4], where *Y_t,k _*is the annual malaria incidence year *t*, and the start month *k *of the twelve-month period was either April (*k *= 4) or September (*k *= 9) [17]. Similarly, the relative change in certain meteorological variable over 12 months periods preceding the malaria periods with a lag of one to three months was represented by Ξ_*t,l,k *_= *X_t,k,l _*- *X*_*t*-1,*k*,*l*_. Malaria was regressed against certain meteorological variable in a first order auto-regressive (AR1) model:

$$\Omega_{t,k}=\varphi_{k}\Omega_{t-1,k}+\beta_{l,k}\left(\Xi_{t,l,k}-\varphi_{k}\Xi_{t-1,l,k}\right)+\varepsilon_{t,k},$$

The Pearson correlation coefficient between (Ω_*t,k *_- *φ_k _*Ω_*t-*1*,k*_) and (Ξ_*t,l,k *_- *φ_k _*Ξ_*t*-1,*l,k*_) was then calculated. Because monthly average temperature, monthly average maximum temperature and monthly average minimum temperature were closely correlated with each other, monthly average maximum temperature was selected and the others were obsolete. The monthly average maximum temperature, monthly relative humidity and monthly rainfall over year *t *were accumulated and averaged as annual average maximum temperature, annual average relative humidity and annual average rainfall.

## Results

### Spearman correlation analysis

Spearman correlation analyses were performed relating monthly malaria incidence to monthly meteorological variable (Table 1). The monthly average temperature, monthly average maximum temperature, monthly average minimum temperature, monthly relative humidity and monthly rainfall were significantly positive-correlated with monthly malaria incidence over the study period. Amoung the meteorological variables, the monthly relative humidity was most closely correlated to monthly malaria incidence (r = 0.543, *P *< 0.01). And the monthly rainfall was least correlated to monthly malaria incidence (r = 0.348, *P *< 0.01). There was closely correlation between different temperature variables. The correlation coefficient for the association between relative humidity and average minimum temperature (r = 0.836, *P *< 0.01) was greater than that for the association between relative humidity and other two kinds of temperatures.

**Table 1:** Spearman correlations coefficient between meteorological variables, untransformed malaria case count and logarithmically transformed malaria case count

	relative humidity	rainfall	average temperature	average maximum temperature	average minimum temperature	malaria incidence
relative humidity	1.000	0.844**	0.794**	0.728**	0.836**	0.543**
rainfall	0.844**	1.000	0.738**	0.675**	0.772**	0.348**
average temperature	0.794**	0.738**	1.000	0.981**	0.990**	0.518**
average maximum temperature	0.728**	0.675**	0.981**	1.000	0.954**	0.529**
average minimum temperature	0.836**	0.772**	0.990**	0.954**	1.000	0.510**
malaria incidence	0.543**	0.348**	0.518**	0.529**	0.510**	1.000

Significance of correlation coefficient different from zero: ** = *P *< 0.01

### Cross-correlation analysis

**Figure 3:**
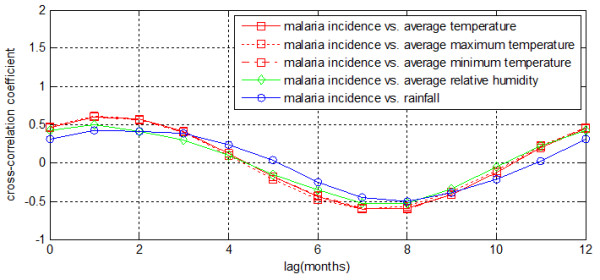
**Cross-correlation coefficients of time series of monthly meteorological variables and monthly malaria incidence at several lags for Motuo County**.

A local maximum cross-correlation between malaria and meteorological variables was found when meteorological variables were preceding malaria by zero to four months (Figure 3). For average temperature, average maximum temperature, average minimum temperature the lag was one month. For rainfall, the lag was two months. The peak correlation coefficient for the association between malaria incidence and each meteorological variables (except rainfall) were as high as 0.5. The correlation coefficient between malaria incidence and monthly rainfall (r < 0.5) was relatively smaller than other two meteorological variables. A local minimum cross-correlation between malaria and each meteorological variable were found when meteorological variables were preceding malaria by five to ten months.

### Cross-correlation analysis with pre-whitening

**Figure 4:**
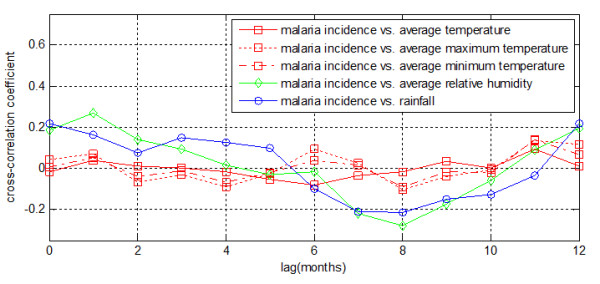
**Cross-correlation coefficients of time series of pre-whitened monthly meteorological variables and monthly malaria incidence time series at several lags for Motuo County**.

For pre-whitening, the model SARIMA(*p *= 1, *d *= 0, *q *= 0, *P *= 2, *D *= 1, *Q *= 2 *p *= 1, *d *= 0, *q *= 0, *P *= 2, *D *= 1, *Q *= 2) was selected. Figure 4 showed the effect of pre-whitening on the malaria incidence time series and meteorological variable time series for Motuo County. With pre-whitened time series, the cross-correlograms looked entirely different from the cross-correlograms without pre-whitening. Correlations were generally weaker with pre-whitening than without. The cross-correlation coefficient for the association between pre-whitened temperature and malaria incidence was fluctuated randomly. Weak positive correlation coefficients were found at lags of zero to two months for pre-whitened relative humidity and rainfall. There was a local maximum at a lag of zero and one month of correlation coefficients for relative humidity and rainfall respectively. Weak negative associations were found at lags of seven to nine months.

### Inter-annual analysis

From table 2, it shows that both differenced annual average relative humidity and differenced annual average rainfall showed significant positive correlations with differenced annual malaria incidence. The significant positive correlation coefficients (r) are 0.451 (*P *< 0.05) and 0.432 (*P *< 0.05) for differenced annual average rainfall and differenced annul average relative humidity respectively. Strong negative correlation coefficient between differenced annual average maximum temperature and differenced annual malaria incidence is significant (r=-0.668, *P *< 0.01).

**Table 2:** Maximum and minimum Pearson product-moment cross-correlation coefficients, starting month and lag (number of months that malaria case time series are lagged behind) for which the maximum or minimum occurred, and significance of the regression coefficient for differenced meteorological variables and differenced annual malaria case time series (n = 22), corrected for first order auto regressive correlation

District	Minimum		Maximum	
	
	r	start month (lag)	r	start month (lag)
Differenced annual average maximum temperature vs. differenced annual malaria incidence	-0.668**	9(1)	0.286	4(3)
Differenced annual average relative humidity vs. differenced annual malaria incidence	-0.382	4(3)	0.432*	9(1)
Differenced annual rainfall vs. differenced annual malaria incidence	-0.207	4(3)	0.451*	9(1)

r = Pearson product moment correlation coefficient

Significance of regression coefficient different from zero:* = *P *< 0.05, ** = *P *< 0.01

## Discussion

The variations in annual incidences indicated that certain factors rather than biological characteristics of parasites determine the transmission of the diseases. Many factors, such as vector species and abundance, human behavior, population immunity, social and economic status and control measures, are known to have significant influence on the transmission of malaria [2,4,22-24]. Meteorological variables are considered as the environmental factors for increased risk of malaria because of their impacts on the Plasmodium incubation rate and mosquito vector activities [25].

Spearman correlation conducted relating monthly incidence of malaria to various monthly meteorological variables. From Table 1, it can be seen that meteorological variables, such as relative humidity, temperatures, rainfall, and malaria incidence showed strong positive significant correlations. Among the meteorological variables, relative humidity and malaria incidence showed the greatest correlation (0.543, *P *< 0.01). The correlation coefficient for the association for rainfall and malaria incidence was 0.348 *(P *< 0.01). The correlation coefficients between temperatures and malaria incidence were slightly smaller than the coefficient between relative humidity and malaria incidence. It was reasonable to conclude that relative humidity influence the activities of mosquitoes directly, such as biting rate and breeding rate. Relative humidity was closely correlated with temperatures and rainfall and it was a result of temperatures, rainfall and other environmental factors.

It has been investigated that temperature and rainfall played determinant role of environmental factors in the transmission of malaria. But the influence was not directly or linearly. But temperature and rainfall may not influence the transmission of malaria in a linear and direct way. Especially the rainfall factor influencing malaria transmission was more complex. The rainfall often leaded to small puddles serving as mosquito breeding sites and increases humidity, which enhanced mosquito survival. However, the relationship between mosquito abundance and rainfall was nonlinear.

Characterizing temporal patterns of clinical malaria provides insights into the important drivers of this disease, including meteorological variables such as rainfall and temperature that influence seasonal patterns that influence long-term trends. Malaria incidence time series and meteorological variable time series have high cross-correlation at short lags and long lags. Positive maximum cross-correlation was observed between malaria and average temperature, average maximum temperature, average minimum temperature and relative humidity at a lag of one month. But this was observed between malaria and rainfall at a lag of two months. Although malaria and meteorological variables showed high cross-correlations in Motuo County, variation from normal monthly malaria incidence patterns showed limited cross-correlation with variation from normal monthly meteorological variable patterns, and therefore meteorological variables may have limited use for predicting malaria.

This study confirmed that temperature had great influence with malaria and malaria responses quickly when temperature was varying. It was understandable that there was usually approximate 1 month period in the course of the malaria infection cycle from wiggler becoming an infectious mosquito to mosquito biting humans and finally human developing malaria symptoms. The correlation coefficient for the association between monthly malaria case count and synchronous relative humidity was smaller than that for the association between monthly malaria case count and relative previous monthly. The correlation coefficient for the association between monthly malaria incidence and monthly average maximum temperature was similar to the correlation for the association between monthly malaria case count and relative humidity. It also implied that malaria prevalence was sensitive to temperature changes. It could be interpreted that relative humidity also has great influence in the life cycle of mosquito and behavior of biting humans. But the correlation coefficient for the association between monthly malaria case count and rainfall is smaller than that of correlation coefficient between other meteorological factors and monthly malaria case count. Rainfall showed complex association with malaria incidence. Rainfall was the least influencing factor with a two-month lag effect, which lag effect is longer than that of temperature and humidity. It could be concluded that rainfall affect the malaria case count through other meteorological and environment. The relationship between mosquito abundance and rainfall was nonlinear. The rainfall often leads to small puddles serving as mosquito breeding sites and increase humidity, which enhances mosquito survival. Abundant rainfall and accumulation of surface water probably wash out or destroy mosquito density.

For pre-whitened series, the cross-correlagrams looked entirely different from the cross-correlograms without pre-whitening. With trend and seasonality were removed by pre-whitening, there is no obvious trend in the cross-correlation coefficient between temperature and malaria incidence (Figure 4). It could be interpreted that the influence of temperature to malaria incidence was direct and it was changing with seasons. Weak positive correlation coefficients were found at lags of zero to two months for pre-whitened relative humidity and rainfall. There was a local maximum at a lag of zero and three month of correlation coefficients for relative humidity and rainfall respectively. Weak negative associations were found at lags of seven to nine months. Rainfall and relative humidity almost had the same influencing direction. Because of affected by rainfall, the cross-correlation coefficient between relative humidity and malaria incidence almost has the same trend as rainfall when trend and seasonality were removed. Because of the short lags when meteorological variables affect malaria transmission, it is difficult to do accurate long-term malaria incidence prediction using meteorological variables. In the cross-correlation analysis with pre-whitening, we can see that rainfall positive correlated to malaria when lag if from 0 to 5 month and negative correlated to malaria when lag is from 6 to 12 months. And this could be taken into account when do coarse malaria prediction or make measures for next year malaria control.

From table 2, it can be seen that both differenced annual average relative humidity and differenced annual average rainfall showed significant positive correlations with differenced annual malaria incidence. Strong negative correlation coefficient between differenced annual average maximum temperature and differenced annual malaria incidence was significant. Inter-annual analysis showed that the changes in meteorological variables have great influence to the malaria incidence. Generally speaking, increasing in rainfall and relative humidity implied malaria incidence increasing. But this was not the case when annual mean temperature changes.

These findings should be considered in future malaria prevention and control projects an in the development of spatiotemporal modes of malaria transmission in Linzhi Prefecture as well. In 2009, Action Plan of malaria elimination was proposed by MOH. So in such situation, how to distribute the recourses effectively is too important for the authorities, especially in the special areas such as Tibet.

## Conclusion

Meteorological variables were important environmental roles in malaria transmission in Motou County. Relative humidity was the greatest influence factors, which affected the mosquito survival directly. The relationship between malaria incidence and rainfall was complex and it was not directly and linearly. The lags of temperature and relative humidity were similar and smaller than that of rainfall. Since the lags of meteorological variables affecting malaria transmission were short, it was difficult to do accurate long-term malaria incidence prediction using meteorological variables. Annual varying effects of meteorological variable like rainfall and relative humidity maybe used to predict malaria.

## Limitations of this study

It was acknowledged that there were likely to have been imperfections in the data given that they were obtained from a passive surveillance system. According to a 2005 national report, it was estimated that only 1/18 (5.6%) cases in China are notified [26] and even that Motuo County was the last accessible county in China, but most villages can only be reached by foot. Malaria diagnosis was also imperfect, and most of malaria cases were diagnosed based on clinical symptoms.

## Conflict of interests

The authors hereby certify that no conflict of interest of any kind occurred in the framework of this study.

## Authors' contributions

FH conducted the analysis and drafted the manuscript, SSZ assisted with the statistical analysis and created figure 1, HJW collected the malaria incidence rate and SSZ and LHT provided extensive and comments on the manuscript. All authors read and approved the final manuscript.
